# Obituary

**Published:** 1883-10

**Authors:** 


### Obituary.

THE LATE MISS DICK.—The death at Burntisland on the evening of Saturday last, at the advanced age of 92, of Miss Mary Dick, sister of the late Professor Dick, founder of the Edinburgh Veterinary College, is an event of some public interest both retrospectively and prospectively. She was intimately associated with her brother in founding and endowing the College, and since his death in 1866, when the management was transferred to the Town Council of Edinburgh, Miss Dick has continued to take the warmest interest in the prosperity of the institution. Under her brother's will, Miss Dick had been constituted residuary legatee. and managed the Burntisland property personally. By her death a large annual revenue from house property and fees will be available for the service of the College, which henceforth may be expected to be placed on a far more satisfactory basis, and to offer still greater advantages for the cultivation of veterinary science.

Miss Dick was born June 1st. 1791 at Whitehorse Close, Edinburgh, where her father, who was a blacksmith, had a forge. Her reminiscences of old Edinburgh were very entertaining. She used to relate that she had been offered the perusal of several of Scott's novels yet only in MS., but had declined to read them both then and ever afterwards. This is doubtless to be explained by the fact that her political and ecclesiastical proclivities lay from the first in a direction diametrically opposed to those of Sir Walter Scott. At the age of twelve she crossed the Firth in an open boat to Kirkcaldy, paying 2s. for her passage, part of which consisted in being carried from the boat to the landing place on the boatman's shoulders. Through her brother she enjoyed a very extensive acquaintanceship both at home and abroad, which she kept up to the last, every day posting and receiving a considerable number of newspapers and correspondence bearing mainly on public questions. She was an ardent Liberal, and advocated female suffrage; greatly satirical on modern extravagance and effeminacy; boasting, for example, that she had never taken a walk for health in her life, and that she had never had a cough. Her funeral takes place to-morrow, when her remains will be interred in the Calton Burying-ground, Edinburgh.—*Scotsman.*

